# Conductive Polyaniline-Based/Polyethersulfone Ultrafiltration Membranes: Morphology, Wettability and Short-Cycle Electrochemical Cleaning

**DOI:** 10.3390/membranes16060194

**Published:** 2026-06-03

**Authors:** Maria Antonia Rodrigues De Paulo, Roger Gonçalves, Ernesto Chaves Pereira, Fernando Henrique Cristovan, Adriana Coatrini Thomazi, José Arnando Costa, Caio Marcio Paranhos

**Affiliations:** 1 Center for the Development of Functional Materials—CDMF, Department of Chemistry, Federal University of Sao Carlos, Sao Carlos 13565-905, SP, Brazil; mariapaulo@estudante.ufscar.br (M.A.R.D.P.); rgoncalves@ufscar.br (R.G.); ernesto@ufscar.br (E.C.P.); 2 Institute of Exact Sciences and Technology, Federal University of Jataí, Jataí 75801-615, GO, Brazil; fhcristovan@ufj.edu.br; 3 National Nanotechnology Laboratory for Agriculture (LNNA), Embrapa Instrumentation, XV de Novembro ST., 1452, Sao Carlos 13560-970, SP, Brazil; adriana.thomazi@embrapa.br; 4 Study Group on Structured Nanomaterials—GENE, Institute of Education Sciences, Federal University of Western Pará, Santarém 68040-070, PA, Brazil; jose.asc@ufopa.edu.br

**Keywords:** electromembranes, polyethersulfone, polyaniline, anti-fouling

## Abstract

Fouling limits the performance and lifetime of polyethersulfone (PES) ultrafiltration membranes. We investigated the effect of blending polyaniline (PAni·DBSA) into PES on membrane morphology, wettability, permeability and antifouling behavior, and we evaluated a simple electrochemical cleaning protocol for fouled membranes. A series of PES/PAni·DBSA membranes with different PAni loadings were characterized by SEM, BET, AFM, contact angle, TGA and porosity analysis. Initial water flux (J), bovine serum albumin (BSA) rejection (RR) and flux recovery ratio (FRR) were measured in a dead-end filtration cell. Electrochemical cleaning was applied to selected fouled membranes, and post-cleaning flux and rejection were measured. PAni·DBSA incorporation produced a hierarchical pore structure and altered near-surface texture. Contact angle decreased from 76° to 54°, and swelling increased for intermediate PAni loadings. Initial pure-water fluxes ranged from 5.9 to 39.3 L·m^−2^·h^−1^. When expressed as absolute percentages, the best performing membrane in terms of reversible fouling recovered 8.12 times of its initial flux. Multivariate analysis indicates that surface hydration and height distribution explain more variance in FRR than Rq alone, consistent with a synergistic role of texture and wettability. Electrochemical treatment substantially increased both flux and rejection for tested membranes, indicating effective foulant mobilization.

## 1. Introduction

Population growth, agricultural expansion, urbanization, and climate change have intensified the global water crisis, leading to an increasing imbalance between water supply and demand [1,2]. In this context, the development of efficient technologies for water and wastewater treatment has become essential to ensure access to safe drinking water [2,3,4]. Conventional treatment processes, such as coagulation–flocculation and sedimentation, have shown limited effectiveness in removing emerging contaminants, including dyes, pharmaceuticals, pesticides, microplastics, and personal care products, which pose risks to both aquatic ecosystems and human health [5,6,7,8,9,10,11].

Pressure-driven membrane processes, including ultrafiltration, microfiltration, nanofiltration, and reverse osmosis, have become well-established technologies for water purification due to their high separation efficiency and operational simplicity [12,13,14]. Consequently, these processes have gained increasing relevance in industrial applications and desalination plants [15,16,17,18]. Polymeric membranes based on materials such as polysulfone (PSf), polyvinylidene fluoride (PVDF), and polyethersulfone (PES) are widely used due to their mechanical strength and chemical stability [19,20,21,22,23,24]. Among them, PES membranes stand out due to their excellent physicochemical properties, although their intrinsic hydrophobicity promotes foulant accumulation and exacerbates fouling phenomena [20,21,22,23,24]. However, membrane fouling remains a major limitation, as it reduces permeate flux, compromises separation performance, and shortens the membrane lifespan [25,26,27]. Fouling can occur through different mechanisms, including pore blocking and cake layer formation, depending on the interactions between foulants and the membrane structure [28,29,30].

To address this limitation, the incorporation of intrinsically conducting polymers (ICP) has emerged as a promising strategy for fouling mitigation [31,32]. Under an applied electric potential, conductive membranes can generate surface charges that promote the electrostatic repulsion of foulants, improving the antifouling performance [33,34]. In this context, electromembranes (EM) combine physical separation with electrochemical phenomena such as electrooxidation, electroreduction, electroadsorption, and electrostatic repulsion [35,36,37]. In addition, membranes incorporating ICP have been investigated in applications such as gas separation and electrodialysis, highlighting their versatility in separation processes [38,39].

Among ICPs, polyaniline (PAni) stands out due to its low cost, environmental stability, ease of synthesis, and tunable physicochemical properties through doping [40,41,42,43]. In addition, the presence of amino functional groups contributes to increased hydrophilicity, which is directly associated with improved antifouling behavior [43,44]. Several studies have explored the use of PAni in membrane systems, particularly in combination with nanomaterials such as graphene-based materials [3,45,46], titanium dioxide [33,47], metal–organic frameworks [48], and other metallic nanoparticles [15,49,50], demonstrating improvements in transport and antifouling performance.

However, despite these advances, there are no reports on the direct incorporation of PAni into PES matrices for membrane fabrication. This gap is particularly relevant, as such an approach may enhance membrane hydrophilicity and reduce interactions with foulants. In this regard, the use of dodecylbenzenesulfonic acid (DBSA) as a dopant for PAni is of great interest, since it can improve electrical conductivity, solubility, and compatibility with polymeric matrices [51,52]. Previous studies have shown that doping PAni with organic acids enhances charge delocalization and conductivity [53,54], while also improving antibacterial activity, which may contribute to biofouling mitigation [55].

Based on these considerations, the central hypothesis of this study is that the direct incorporation of DBSA-doped PAni into a PES matrix can enhance membrane hydrophilicity, improve antifouling behavior, and introduce additional functionalities, resulting in improved filtration performance. Therefore, this study aims to develop EMs based on PES and PAni using the phase inversion method, and to systematically evaluate the effect of PAni incorporation on membrane properties, filtration efficiency, and fouling resistance. In addition, we systematically investigate a practical electrochemical cleaning and regeneration protocol for these EMs, not addressed in prior nanoparticle-based modifications, was evaluated.

## 2. Materials and Methods

### 2.1. Materials

PES was obtained from Solvay Inc. (Paulinia, Brazil) (Veradel HC A-301 NT, $\bar{M}w$ = 15,000 gmol^−1^). Aniline, ammonium persulfate (APS), DBSA, and bovine serum albumin (BSA, $\bar{M}w$ = 66 kDa, prepared in buffer solution pH 7.2) were purchased from Sigma Aldrich (Sao Paulo, Brazil). Hydrochloric acid (HCl, 37% conc.), ammonium hydroxide (NH_4_OH), and N-methyl-2-pyrrolidone (NMP) were acquired from Synth (Diadema, Brazil). Aniline was purified by vacuum distillation. Other reagents were utilized without further purification.

### 2.2. Synthesis of PAni.DBSA

PAni was prepared by chemical oxidative polymerization. First, 0.02 mol of aniline was dissolved in 150 mL of HCl (1 mol L^−1^), and an oxidizer solution was prepared with APS and HCl (1 mol L^−1^) solution. The monomer:oxidizing agent ratio that was used was 4:1 mol:mol. This proportion was selected due to a significant improvement in the electrical conductivity of the material [39,56,57]. Subsequently, 150 mL of a 0.005 mol APS solution was quickly introduced into the aniline precursor. The reaction was kept under static conditions at 5 °C for 3 h. The PAni was washed with HCl (0.1 mol L^−1^) and deionized (DI) water. Then, the PAni was dried for 24 h at 60 °C, and the emeraldine salt (PAni-ES) was obtained.

**Figure 1:**
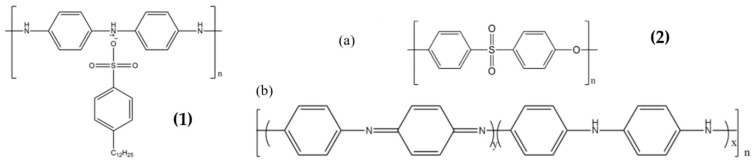
(**1**) Molecular structure of PAni.DBSA; (**2**) molecular structure of: (**a**) PES and (**b**) PAni (y is the oxidation state, and x is 1-y).

The conversion of PAni-ES to PAni-EB was performed by dispersing 100 mg of the salt in a 100 mL of 0.1 mol L^−1^ NH_4_OH solution. After 24 h of continuous stirring, the suspension was vacuum filtered, washed thoroughly with distilled water, and dried under controlled temperature (40 °C) for 24 h. The EB was doped with DBSA (Figure 1(1)), solubilizing the PAni in DBSA in the proportion 1:3 (mol:mol) and was left stirring for 48 h.

### 2.3. Membrane Preparation

The PES and PES/PAni.DBSA membranes were prepared by the nonsolvent induced phase separation method (NIPS), based on the methodology described by Khan et al. [58]. The PES was dried in a vacuum oven at 150 °C for 3 h to remove adsorbed moisture; then it was solubilized (20:80 *w*:*w*) in NMP, under magnetic stirring, for 48 h at 70 °C. For the preparation of the conductive membranes, PAni was previously dispersed in NMP for 48 h and poured into the PES solutions under magnetic stirring for 48 h at 50 °C. Different PAni.DBSA concentrations by weight of PES were incorporated, as presented in Table S1. The PES/PAni.DBSA dispersions were kept in an ultrasonic bath for 10 min to eliminate bubbles and then poured onto a glass plate and spread using a 150 µm extensometer. The plate was then submerged in deionized water for 48 h. Fresh water was replaced every 24 h during the phase separation. The membranes were then dried at room temperature.

### 2.4. Membrane Characterization

FTIR spectra were recorded using a Bruker Vertex 70 infrared spectrophotometer equipped with an attenuated total reflectance (ATR) accessory (Billerica, MA, USA). Scans were acquired from 4000 to 400 cm^−1^, with 32 scans and a 4 cm^−1^ resolution of transmission mode.

UV–Vis absorption spectra were measured on a Cary 700 spectrophotometer (Melbourne, Australia), using a diffuse reflectance accessory to probe interactions within the membranes. Measurements were performed at room temperature (25 °C) over the 200–800 nm range.

X-ray diffraction patterns were collected at room temperature with a Rigaku SmartLab SE (Yamanashi, Japan) diffractometer using Cu Kα radiation (λ = 1.5418 Å) operated at 40 kV and 30 mA. Scans were performed over 2θ = 5–80° at a scan rate of 2°. min^−1^.

Membranes surface and cross-sectional morphologies were examined by scanning electron microscopy (SEM) (FEI Magellan 400 L, Eidhoven, The Netherlands). For cross-sectional imaging, samples were immersed in liquid nitrogen and then fractured prior to observation.

Specific surface area measurements were performed on an ASAP 2020 instrument (Micrometrics, Norcros, GA, USA). A membrane sample of approximately 0.13 mg was finely cut and placed in a glass sample holder. Prior to analysis, the sample was degassed under vacuum at 10 µm Hg and 40 °C for 12 h to remove physiosorbed species. After degassing, the sample was reweighed and analyzed using nitrogen at 77 K. The specific surface area was calculated using the Brunauer–Emmett–Teller (BET) method, using adsorption data in the relative pressure (P/P_0_) = 0.05–0.30.

Thermal stability of the membranes was evaluated using a thermogravimetric analyzer (TGA, Netzsch 209 F3 Tarsus, Selb, Germany). Samples were heated from 20 to 800 °C, at 10 °C.min^−1^ in a N_2_ atmosphere.

The electrical conductivity (σ) of membranes was measured using four-probe method at room temperature and calculated by

(1) $$\sigma =\frac{I\cdot L}{V\cdot A}$$

where I is the electrical current, L the distance between probes, V the voltage and A the cross-sectional contact area.

The surface morphology and roughness were quantified by Atomic Force Microscopy (Bruker AFM, Santa Barbara, CA, USA) in tapping mode (silicon cantilevers, ~190 kHz) over 10 µm × 10 µm areas at three distinct non-overlapping locations per membrane. The scan resolution was 512 × 512 pixels, and the scan rate was 0.5–1.0 Hz. Raw images were processed in Gwyddion (version 2.70) using first-order flattening and line correction; no additional filtering was applied. The surface parameters, Rq, Ra, skewness (Rsk) and excess kurtosis were computed per area and reported as the mean ± standard deviation. The Gwyddion macro and representative raw images are provided in the Supplementary Information.

The surface hydrophilicity was assessed by contact angle measurements and by determining the swelling degree. Contact angles were measured with a Ramé-Hart 260 F4 goniometer (Netcong, NJ, USA), using distilled water as the probe liquid by the sessile drop method. Measurements were performed in triplicate for each film, yielding nine measurements per sample. The swelling degree (SD) was determined as follows: samples (2 × 2 cm^2^) were weighed dry (DM), immersed in 50 mL of distilled water at room temperature for 24 h, and then weighed after removing excess surface water with filter paper (WM). The swelling degree was calculated as

(2) $$SD\left(\%\right)=\frac{\left(WM-DM\right)}{DM}\cdot 100$$

### 2.5. Performance Evaluation and Anti-Fouling Studies

The permeability was evaluated by measuring the pure water flux (J) using a dead-end laboratory-scale filtration cell (50 mL volume) with an effective membrane area of 8.0 cm^2^. Membranes were soaked in distilled water for at least 48 h prior to test to ensure a steady permeation flux. The flux was calculated as

(3) $$J=\frac{V}{A\cdot t}$$

where J is the deionized water flux (L·m^−2^·h^−1^), V is the permeated volume (mL), A is the membrane area (cm^2^), and t is the permeation time (h). The transmembrane pressure was maintained at 2.0 bar using N_2_ flow.

The antifouling performance was assessed following the method described by Costa et al. [59]. The initial pure water flux was recorded as Jw1. A BSA aqueous solution (0.5 g·L^−1^ in pH 7.2 buffer solution) was then filtered through the membrane for 1 h. After fouling, the membrane was removed from the cell, rinsed with deionized water, and then reinstalled to measure the pure water flux Jw2. The flux recovery ratio (FRR) was calculated as [60]

(4) $$FRR=\frac{Jw2}{Jw1}\cdot 100$$

where Jw2 is the water flux measured after exposure to BSA. BSA concentrations in the permeate were measured by UV spectroscopy at 280 nm to determine retention. The BSA retention rate (RR) was calculated as

(5) $$\%RR=(1-\frac{Cp}{Cf})\cdot 100$$

where Cp and Cf are the BSA concentrations in the permeate and feed, respectively.

Electrochemical cleaning was performed in a 250 mL beaker containing 150 mL 0.5 mol L^−1^ NaCl electrolyte. The setup comprised an external DC power supply connected to two electrodes: the fouled membrane (working electrode) and a 10 cm^2^ Pt-Ti counter electrode [61], without the use of a reference electrode, and the distance used between the electrodes was 5 cm. For the PES membrane (M1), which is electrically insulating, no direct electron conduction occurs through the membrane; in this case, the applied potential is transmitted through the electrolyte and current collector configuration. Each fouled membrane was subjected to one of two polarization treatment in separate experiments: anodic polarization at +2 V for 10 min or cathodic polarization at −2 V for 10 min, without stirring. This protocol was chosen to compare the predominantly oxidative cleaning mechanism at the anode with the reductive/alkaline-generating mechanism at the cathode [62,63]. No external electrical field was applied during the filtration experiments. Electrochemical cleaning was performed only after filtration. After electrochemical treatment, the membranes were rinsed, and the water flux, permeate purity, and the amount of collected foulant were re-evaluated.

## 3. Results

### 3.1. Characterization of Membranes

Figure S1 presents the FTIR spectra of the PES and PES/Pani.DBSA membranes. The para-substituted aromatic ring of PES shows characteristic C-H bending bands at 839 cm^−1^ and 1011 cm^−1^, while absorptions attributed to the sulfone group appear in the 1149–1300 cm^−1^ region. Characteristic bands of PAni are not apparent in the membrane spectra (Figure S2), most likely because of the low PAni content. No appreciable band shifts or the emergence of new bands are observed upon the incorporation of PAni, which indicates that interaction between PAni and PES is predominantly physical (non-covalent) rather than chemical. For reference, the expected PAni bands occur near 3439 cm^−1^ (N-H stretching), 1567 cm^−1^ (quinonoid ring stretching, N-Q-N), and 1479 cm^−1^ (benzenoid ring stretching, N-B-N) [44]. The molecular structures of PES, PAni and PAni.DBSA are shown in Figure 1. Because of the low PAni content and the limited sensitivity of FTIR for detecting dispersed conductive polymer in this matrix, diffuse reflectance UV–Vis spectroscopy is the more appropriate technique to confirm the presence of PAni.DBSA.

In the UV–Vis spectra of the membranes containing PAni.DBSA (Figure S3), a broad absorption band centered at ~600 nm appears and grows in intensity with increasing PAni.DBSA content. This feature is attributed to the polaron band and is absent in the pristine membrane (M1), confirming that PAni.DBSA is present in its conductive (doped) form within the membrane. A second absorption near 350 nm is observed and assigned to π-π* transitions of benzenoid rings [45]. Pristine PES exhibits an absorption at ~315 nm, which arises from π-π* transitions of the benzene units in the polymer backbone; the intensity of the benzenoid-related bands increases as the PAni.DBSA content in the membranes rises.

The XRD patterns of the prepared membranes (Figure S4) show no sharp diffraction peaks, consistent with a predominantly non-crystalline structure; this is expected because PES is an amorphous polymer. Instead of discrete peaks, a broad diffuse hump is observed between 14.5° and 26.5° (2θ), which reflects a limited short-range ordering or a poorly defined crystallizable fraction within the polymer matrix. The diffraction patterns of the PAni-modified membranes closely resemble that of the pristine PES, indicating that the incorporation of the conductive polymer does not produce significant changes in the long-range structural organization of the matrix. This behavior can be attributed to the low content and to the intrinsically amorphous character of PAni.DBSA; so, no new crystalline domain is formed upon blending [64,65].

**Figure 2:**
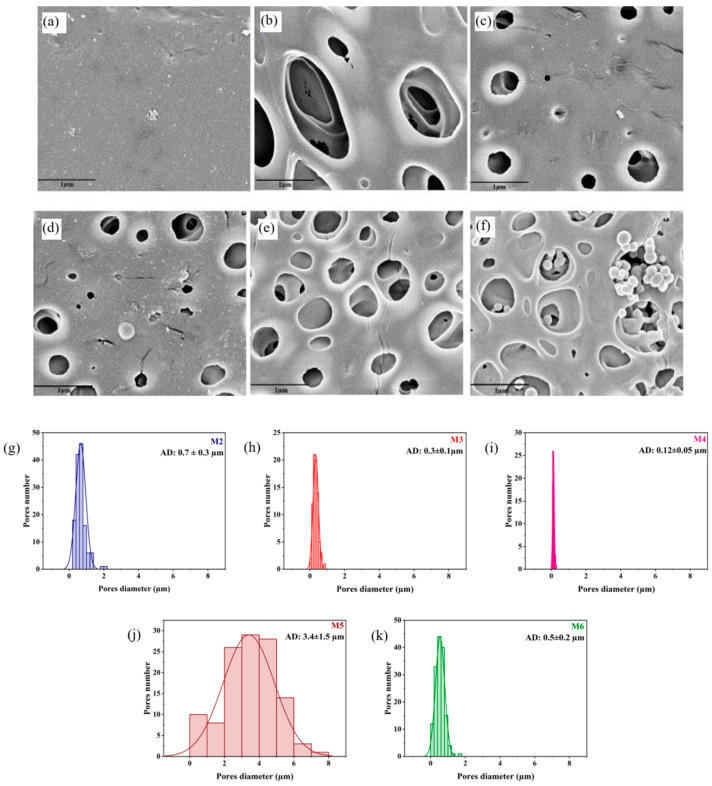
Scanning electron microscopy (SEM) surface images of (**a**) M1; (**b**) M2; (**c**) M3; (**d**) M4; (**e**) M5; and (**f**) M6 and pore diameter analysis of (**g**) M2; (**h**) M3; (**i**) M4; (**j**) M5, and (**k**) M6.

Figure 2 shows representative SEM micrographs along with the corresponding pore size distribution histograms for pristine and PAni-modified PES membranes. Discrete well-dispersed clusters, attributed to PAni.DBSA domains [47], are observed on the membrane surfaces. Compared to the pristine PES membrane (M1), the modified membranes (M2–M6) exhibit a higher surface pore density and a broader pore size distribution at the same magnification. The average pore diameters, determined using ImageJ (version 1.54k), are as follows: M2 = 0.67 µm, M3 = 0.43 µm, M4 = 0.12 µm, M5 = 3.4 µm, and M6 = 0.50 µm. These morphological features are consistent with structures formed via the NIPS process, as well as with the tendency of PAni.DBSA to locally aggregate at higher concentrations [17]. The observed increase in surface porosity is expected to enhance water permeability. Additionally, membranes exhibiting smaller pore diameters may provide improved selectivity, potentially reducing the passage of fouling agents.

The specific surface area and pore-related properties of the membranes were further investigated using the BET method based on nitrogen physisorption (Table 1).

BET nitrogen physisorption yields modest specific surface areas (19.7–24.7 m^2^·g^−1^) and pore volumes (0.07–0.11 cm^3^·g^−1^). M3 exhibits the highest surface area (24.73 m^2^·g^−1^) and M5 the largest pore volume (0.11 cm^3^·g^−1^). The adsorption–desorption isotherms show hysteresis consistent with Type IV behavior, indicating the presence of meso-/micro-scale porosity accessible to N_2_ (Figure S5) [66,67]. It is important to emphasize that SEM and BET probe different structural domains: SEM resolves µm-scale surface and through-pore morphology, whereas BET quantifies internal surface area and nm-scale pores. The apparent discrepancy between µm-scale SEM pore diameters and the Type IV BET signature is resolved by recognizing a hierarchical porous architecture in these membranes: (i) a macroporous scaffold and through-pores formed by phase inversion (µm scale) that dominate hydraulic permeability and (ii) a finer nm-scale pore network and textured surface that control adsorption, surface area, and short-range foulant interactions [46].

**Figure 3:**
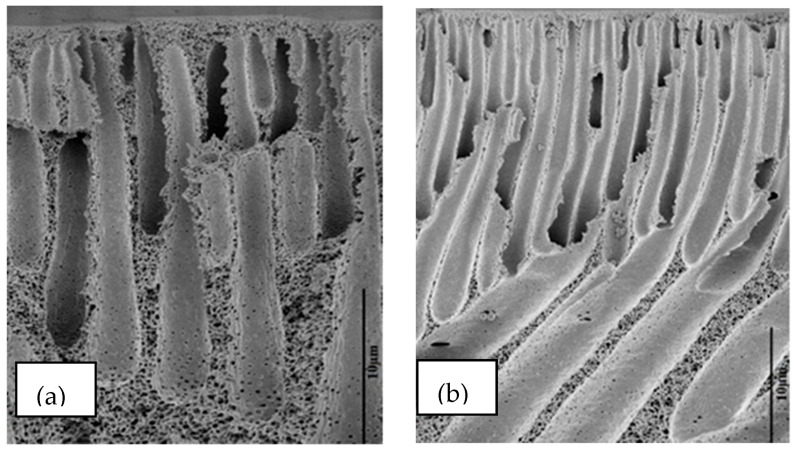
SEM cross-sectional images of (**a**) M1 and (**b**) M3.

Cross-sectional SEM images of M1 and M3 (Figure 3) confirm the anisotropic structure typical of NIPS membranes: a thin selective top layer over a porous substructure with finger-like macrovoids [46]. Incorporation of PAni.DBSA modifies phase-inversion kinetics and thus selective-layer thickness and pore depth; for example, M3 shows a thinner upper selective layer and a higher density of deep pores relative to M1, which can explain its higher permeability while maintaining selective rejection.

The morphological changes induced by PAni.DBSA are consistent with the role of hydrophilic additives in accelerating solvent–nonsolvent exchange during NIPS and modifying demixing pathways [37]. At low PAni.DBSA loadings (M3), there is improved solvent exchange and better dispersion of the doped polymer favor formation of interconnected macrochannels and an exposed near-surface microporous network; at higher loadings, PAni.DBSA aggregation can occur, promoting partial pore blockage and selective-layer irregularities that reduce effective through-pore connectivity. The finger-shaped pores observed in both control and modified membranes reflect the high affinity between NMP and water (the non-solvent), which drives rapid exchange and macrovoid formation during phase inversion [36].

TGA and its derivative (DTG) were used to compare the thermal decomposition behavior of pristine and PAni-modified PES membranes (Figure S6). The onset temperature and DTG peak temperatures for each sample are summarized in Table 2; for the unmodified PES (M1), the principal mass-loss event begins near 519 °C, and the sample retains approximately 38% of its initial mass at the end of the run [68]. Modified membranes show variations in the temperatures and magnitudes of mass-loss steps: M5 and M6 exhibit higher apparent onset/DTG peak temperatures (T_onset_ ≈ 542 °C and 540 °C, respectively) and larger final residues than some lower-loaded samples. These differences indicate that PAni.DBSA incorporation alters the thermal decomposition profile of the composite membranes. Small shifts in T_onset_ or T_max_ can result from the decomposition of the self-associated hydroxyl group (-OH). The mass loss that occurs between 543 and 540 °C in membranes M5 and M6, respectively, is attributed to the decomposition of the PES structure. The increase in the thermal stability with the higher content of PAni.DBSA is evidenced by T_50%_ (M5 and M6 membranes do not decompose to that level) and T_max_ values. T_max_ was calculated form the onset temperature associated with the DTGA peak. Recent investigations have demonstrated an increase in the thermal resistance of membranes containing increasing levels of PAni [69]. The observed increase in T_onset_ for M5 and M6 relative to pristine PES is consistent with an interfacial and barrier-type stabilization. π–π interactions and weak hydrogen bonding between PAni aromatic chains and the PES backbone can raise the energy required to initiate chain scission, while the conjugated aromatic phase can promote early carbonaceous residue/char formation under N_2_ atmosphere that physically retards volatile release and delays apparent mass loss [70,71]. Additionally, the DBSA dopant and the altered microstructure introduced by PAni can change the decomposition pathways and kinetics, producing a higher apparent T_onset_ and larger final residue, even though neat PAni decomposes at lower temperatures [72].

AFM topographies (10 µm × 10 µm scans; Figures S7 and S8) reveal systematic PAni-dependent changes in surface texture. The pristine PES (M1) shows a relatively uniform surface, whereas modified membranes present distinct morphologies: M2 displays a nodular “brain-like” texture; M3 exhibits broad mountain-like features with the largest peak-to-valley amplitudes; M4 is comparatively smooth; M5 shows moderate heterogeneity; and M6 is characterized by sharp isolated peaks. Three-dimensional reconstructions corroborate these observations and clarify the vertical scale of features (z range reported per image). Quantitative metrics (Table S2) show that Rq increases from 6.78 ± 1.2 nm (M1) to 18.1 ± 1.8 nm (M3), while Ra, Rq/Ra, skewness (Rsk) and excess kurtosis provide complementary information on the texture symmetry and peakedness. For example, M3’s high Rq and positive excess kurtosis indicate a broad height distribution with pronounced peaks and valleys, whereas M2’s negative skewness indicates a valley-dominated profile.

### 3.2. Hydrophilicity

**Figure 4:**
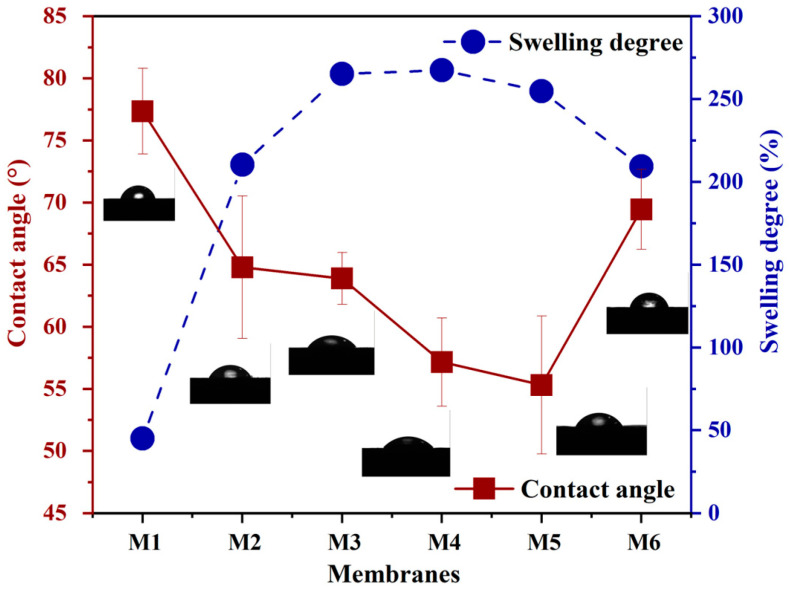
Static water contact angle and swelling degree for membranes.

The contact angle and swelling degree were measured to evaluate the surface wettability and water uptake; the results are shown in Figure 4. All PAni-modified membranes exhibit lower static contact angles than the pristine PES control (M1), indicating increased surface hydrophilicity. The contact angle decreases from 76 ± 4° for M1 to values as low as 54 ± 5° for M5. The swelling degree follows the same general trend for intermediate PAni.DBSA loadings, with a maximum swelling observed for M4 and a decline at the highest PAni.DBSA content (M6). The observed increase in wettability is consistent with the presence of DBSA as a surfactant and with prior reports that PAni incorporation can enhance membrane affinity for water [48].

These wettability changes must be interpreted together with the morphology and surface texture. Cross-sectional SEM images show that selective-layer thickness and through-pore connectivity vary across samples and therefore control bulk hydraulic conductance. AFM demonstrates that PAni.DBSA also modifies the near-surface texture, with M3 showing the largest Rq and a leptokurtic height distribution. The results obtained for the contact angles are consistent with the roughness measurements, where the rougher membranes, such as M3, showed lower contact angle values than the smoother membranes. Finally, although hysteresis effects cannot be ruled out, the roughness results obtained by AFM and the static contact angle measurements provide a reliable interpretation of the wettability behavior of the membranes. Taken together, the data indicate that surface hydration (contact angle and swelling) and local topography (AFM metrics) jointly influence protein–surface interactions: hydrated textured surfaces favor weaker and more reversible BSA adsorption, while valley-dominated textures or large macropore entrances promote convective trapping and irreversible pore blocking under dead-end filtration. These interpretations are consistent with the BET and SEM observations that M3 has an increased specific surface area and a distinct pore distribution compared with M1.

### 3.3. Anti-Fouling Properties of Membranes

**Figure 5:**
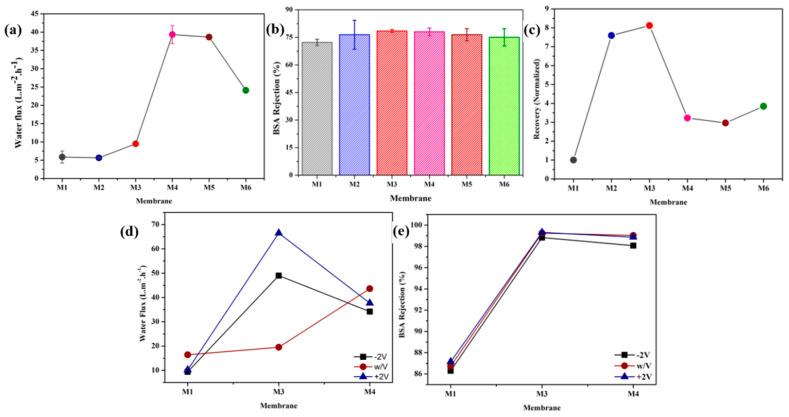
(**a**) Water flux of membranes; (**b**) BSA rejection of membranes; (**c**) recovery rate of the membranes; (**d**) water flux of membranes after electrochemical cleaning; (**e**) BSA rejection of membranes after electrochemical cleaning.

The water flux, BSA rejection and flux recovery were measured in dead-end filtration using a 0.5 g·L^−1^ BSA feed; the results are summarized in Figure 5 and Table 3 and Table 4. The pristine PES control (M1) exhibited a low initial water flux of 5.9 ± 0.4 L·m^−2^·h^−1^, whereas PAni-modified membranes showed higher fluxes (M2: 5.6 ± 0.3; M3: 9.5 ± 0.6; M4: 39.3 ± 2.1; M5: 38.6 ± 2.0; M6: 24.1 ± 1.5 L·m^−2^·h^−1^). These differences reflect the combined effects of selective-layer thickness, macropore connectivity (SEM), and near-surface texture (AFM). The produced membranes exhibited moderate BSA rejection across the set (72–78%); M3 and M4 showed the highest mean rejections (78.5 ± 1.2% and 78.1 ± 1.0%, respectively), while M1 had 72.3 ± 1.5% (Figure 5b).

The flux recovery ratio (FRR, Table 3) was normalized to the pristine PES membrane (M1) to emphasize the relative cleaning efficacy of each PAni-modified membrane independent of large differences in baseline permeability (from ~5.9 to ~39.3 L·m^−2^·h^−1^ as presented above). Normalization facilitates direct comparison of how each membrane responds to the same electrochemical cleaning protocol by removing the confounding influence of architecture-driven flux variability. Absolute FRR (%) values for all samples are provided in Table S4. M3 displayed the highest FRR, indicating that after the applied cleaning protocol the membrane recovered approximately eight times of its initial flux.

The electrical conductivity of the PAni.DBSA membranes was lower when compared to the pristine PES (Table S3). The presence of PAni at low content within the PES matrix promotes a discontinuous bulk conductive network [2,73]. Considering the combined AFM, SEM, contact-angle and swelling results, PAni.DBSA is predominantly surface-localized, producing a thin discontinuous conductive/electroactive layer that alters roughness, wettability and local swelling without forming a continuous bulk percolating network [30,74]. Therefore, the observed improvements in transport and fouling mitigation result from surface conductivity/electrochemical activity, rather than from an increase in through-thickness bulk conductivity.

However, it is important to emphasize that, over time, the fouling process is inevitable. To mitigate the membrane’s fouling, an electrochemical cleaning process was used to assess the possibility of reusing these membranes in a new water treatment cycle. To evaluate the reuse of the membranes, they were subjected to water flux and fouling agent rejection tests, which can be seen in Figure 5. Additionally, different potentials, −2 V, without volt (w/V), and +2 V, and their respective influences on the membrane performance were evaluated. The experimental design involved evaluating anodic and cathodic polarization as two separate and independent treatments and not as sequential steps applied to the same membrane sample. The primary reaction driving the anodic cleaning is oxygen evolution:

$$2H_{2}O\left(l\right)\to \;O_{2}\left(g\right)+4H^{+}\left(aq\right)+4e^{-}$$

which can degrade organic foulants. The applied potential of +2 V is not considered sufficient to cause the irreversible over-oxidation of the PAni.DBSA. This was confirmed by FTIR, where it was not possible to observe a degradation effect, as the characteristic bands referring to the polaronic states did not undergo significant modifications after the treatment. For cathodic cleaning, the primary reaction is hydrogen evolution:

$$2H_{2}O\left(l\right)+2e^{-}\to \;H_{2}\left(g\right)+2OH^{-}(aq)$$

where the local increase in pH can dissolve or dislodge organic and biological foulants [75]. The negative potential is not reported as a significant problem for conductive polymers, as under these conditions, they are found in their stable, fully reduced, and insulating state (leucoemeraldine) [76]. In both cases, the mechanical action of the gas bubbles also contributes significantly to detaching the fouling layer [62].

To quantify the foulant removal we measured the protein concentration in the cleaning solution and in permeate samples; the results are reported in Table 4 as the mass of BSA removed (mg·cm^−2^). These data show that electrochemical cleaning mobilizes a substantial fraction of the adsorbed BSA for PAni-modified membranes (M3 and M4), consistent with their higher FRR. Reporting the mass removed avoids relying solely on flux recovery as a proxy for cleaning efficiency.

After electrochemical cleaning, both the flux and rejection improved for the tested membranes (Table 4). For M3, J increased from 9.5 ± 0.6 to 66.5 ± 3.2 L·m^−2^·h^−1^ after +2 V treatment, and RR increased to 99.3 ± 0.4%. For M4, the flux J decreased slightly from 39.3 ± 2.1 to 37.7 ± 2.0 L·m^−2^·h^−1^ while the rejection ratio increased to 98.9 ± 0.5%. Under cathodic conditions (−2 V), the flux further decreased to 34.2 L·m^−2^·h^−1^, and RR increased to 98.07 ± 0.5%, which indicates the limited recovery of membrane permeability. These results suggest a permeability–selectivity trade-off, possibly associated with fouling layer compaction or pore constriction induced by the electrochemical treatment [20,77,78]. These improvements indicate effective foulant removal and/or cake disruption under the tested conditions. An observed exception is that the M3 membrane with low initial permeation showed the least rejection after cleaning in a subset of experiments. Two plausible explanations are as follows: (i) selective-layer thinning or pore-opening during cleaning (mechanical bubble action or electrochemical effects) increased the flux but reduced the size exclusion, and (ii) removal of a surface cake that had acted as a secondary filter increased the flux but lowered the apparent rejection. The increase in rejection after electrochemical cleaning suggests a reduction in pore size and, consequently, in the apparent Molecular Weight Cut-Off (MWCO), probably associated with interfacial modifications and the presence of a residual selective layer in the M3 membrane.

When combined with the hydrophilicity data, the AFM texture metrics indicate that surface hydration and local topography jointly determine fouling reversibility. An increased Rq together with a reduced contact angle and higher swelling at intermediate PAni.DBSA loadings point to a more strongly hydrated heterogeneous surface that favors weak reversible BSA adsorption rather than irreversible binding. This interpretation explains the observed performance: membranes such as M3, which combine elevated Rq, leptokurtic height distributions and low contact angle, provide many hydrated easily desorbable adsorption sites and therefore show superior FRR and BSA rejection; by contrast, membranes dominated by large macropores or a thinner selective layer (e.g., M4/M5) deliver high initial flux but promote rapid convective pore blocking under dead-end operation, while excessive PAni.DBSA (M6) can cause agglomeration that reduces the effective surface hydration and degrades both flux and recovery. Because the fouling tests were performed in a dead-end cell with 0.5 g·L^−1^ BSA, convective delivery of protein to pore mouths is amplified, and thus, the trade-off between permeability and FRR reflects the coupled action of macropore geometry and nm-scale surface hydration/texture.

From the data shown in Table 4, it is possible to suggest that the foulants are more susceptible to electrochemical oxidation, which facilitates their detachment or degradation during the cleaning process. Nevertheless, the NaCl electrolyte also plays an important role in contributing to the cleaning process. Since all membranes were subject to 30 min immersion prior to the electrochemical cleaning process, the presence of the salt can achieve an ion-exchange reaction with the BSA, causing the collapse of the foulant gel onto the membrane surface, resulting in the increase of RR and J even in the absence of applied voltage. A similar effect was described by Corbáton-Báguena et al. [79] and Tanudjaja et al. [80].

Finally, the membrane that demonstrated the best antifouling behavior was membrane M3, containing only 0.2% PAni.DBSA, which obtained good value before and after electrochemical cleaning. When compared to similar membrane previously reported in literature (Table 5), the present investigation showed the feasibility of incorporating PAni.DBSA to improve the antifouling performance of PES-based EMs.

Table 5 reports both as-prepared and post-electrochemical-cleaning values where available. Because many literature reports present only the as-prepared performance, direct numerical comparisons should be interpreted with caution. We include post-cleaning values to illustrate the regeneration potential of PAni·DBSA-modified PES EMs operational advantage (rapid flux and rejection recovery) as a key outcome of the present study and complements conventional as-prepared metrics.

## 4. Conclusions

Incorporating DBSA-doped PAni into PES membranes alters the phase-inversion morphology, producing a hierarchical pore architecture and modified near-surface texture that together increase wettability and influence fouling behavior. Among the formulations tested, M3 (0.2% PAni·DBSA) delivered the most favorable combination of properties: increased surface roughness with a leptokurtic height distribution, reduced contact angle, improved BSA rejection (~78.5%), and the highest flux recovery after cleaning (FRR ≈ 81%). Electrochemical cleaning in 0.5 M NaCl at ±2 V for short durations effectively mobilized foulants and substantially increased the post-cleaning flux and rejection for selected membranes (e.g., M3 post-cleaning up to 66.5 L·m^−2^·h^−1^, RR ≈ 99%), demonstrating that conductive modification can enable active cleaning strategies. These findings justify further targeted studies—particularly long-term, cross-flow and real-effluent experiments—before asserting practical viability for wastewater treatment or commercial deployment.

## Figures and Tables

**Table 1:** Surface area, pore volume, and pore size of the membranes.

Sample	Surface Area (m^2^·g^−1^)	Pore Volume (cm^3^·g^−1^)	Pore Diameter Range (µm)
M1	22.40	0.07	
M2	21.04	0.07	0.70
M3	24.73	0.10	0.30
M4	20.97	0.08	0.12
M5	23.75	0.11	3.4
M6	19.71	0.09	0.50

**Table 2:** Temperature profile of percent weight loss of PES and PES/PAni membranes.

Sample	T_onset_ (°C)	T_50%_ (°C)	T_max_ (°C)	Residue (%)
M1	519.2	557.48	562.5	37.96
M2	511.5	560.35	522.4	28.51
M3	515.6	560.61	555.0	32.30
M4	512.4	565.23	552.5	35.21
M5	542.6	619.53	556.5	49.64
M6	540.5	--	556.2	67.87

The experimental error associated with the thermal analysis experiments are ±1 °C.

**Table 3:** Membrane properties: Water flux (J), rejection ratio (RR) and flux recovery ratio (FRR) for different membranes.

Sample	J (L·m^−2^·h^−1^)	RR (%)	FRR *
M1	5.9	72.30	1
M2	5.6	76.46	7.59
M3	9.5	78.50	8.12
M4	39.3	78.05	3.22
M5	38.6	76.47	2.96
M6	24.1	75.08	3.85

* The values of FRR were normalized in terms of the pristine membrane M1.

**Table 4:** Membrane properties: water flux (WF) and rejection ratio (RR) for membranes pre- and post-electrochemical cleaning.

Pre-Cleaning			Voltage	Post-Cleaning	
Sample	J (L·m^−2^·h^−1^)	RR (%)	Voltage (V)	J (L·m^−2^·h^−1^)	RR (%)
M1	5.9	72.30	−2	9.4	86.30
			+2	10.2	87.15
M3	9.5	78.50	−2	49.0	98.83
			+2	66.5	99.32
M4	39.3	78.05	−2	34.2	98.07
			+2	37.7	98.87

**Table 5:** Antifouling performance of some previously investigations on PAni-based membranes.

Type of PAni	Membrane Matrix	Measurement Condition	J (L·m^−2^·h^−1^)	FRR (%)	RR (%)	Ref
Zwitterionic PANi	PVDF and sulfonated PVDF (a)	as-prepared	100–160	~100	90–95	[81]
Sulfonated PANi	PSf (a)	as-prepared	170–210	75–85	90–95	[17]
PANi/PMA nanoparticles	PES (a)	as-prepared	270–390 (b)	60.6–77.2	94.3–99.0	[82]
PAni nanofiber	Commercial PSf	as-prepared	40–110	-	98.8–99.2	[43]
PAni nanofiber	PSf (a)	as-prepared	70–170	43.2–70.8	92.0–96.0	[42]
PAni.DBSA	PES (a)	post-cleaning	19–66.5	1–8.12 (c)	98.1–99.6	This study

PVDF = poly(vinylidene fluoride); PSf = polysulfone; PMA = phosphomolybdic acid; (a) phase inversion technique; (b) at 60 min operation time; (c) after electrochemical cleaning.

## Data Availability

The data presented in this study are available from the corresponding author upon request due to privacy concerns.

## Supplementary Materials

The following supporting information can be downloaded at https://www.mdpi.com/article/10.3390/membranes16060194/s1, Table S1: Membrane code and composition. Table S2: Surface roughness and topography parameters obtained from AFM analysis. Table S3: Electrical conductivity of PES and PES/Pani membranes. Table S4: Absolute FRR values obtained for PES and PAni-based membranes. Table S5: Data correlation between several aspects of the membranes. Figure S1: FTIR spectrum of the electromembranes. Figure S2: FTIR spectrum of PAni. Figure S3: UV-vis spectra of the membranes: (***─***) PES; (***─***) M2; (***─***) M3; (***─***) M4; (**─**) M5; (**─**) M6. Figure S4: XRD patterns of membranes. Figure S5. Nitrogen adsorption-desorption isotherms for membranes: (a) M1; (b) M2; (c) M3; (d) M4; (e) M5; (f) M6. Figure S6: Thermogravimetric analysis for membranes: (**─**) M1; (**─**) M2; (**─**) M3; (**─**) M4; (**─**) M5; (**─**) M6. Figure S7: AFM 2D topographies (10 µm × 10 µm). z-range: M1 0–103 nm; M2 0–80 nm; M3 0 – 299 nm; M4 0 – 165 nm; M5 0 – 117 nm; M6 0 – 175 nm; N = 3 areas per sample.” Figure S8: 3D topographical AFM images (10 µm x 10 µm) of the membrane surfaces. Images correspond to samples (a) M1, (b) M2, (c) M3, (d) M4, (e) M5, and (f) M6. The z-axis scale indicates the height range for each sample. Figure S9: (a) Comparison of Rq and Ra values for samples, (b) Average surface line profiles extracted from the AFM topographical images, illustrating the characteristic cross-sectional texture of each membrane. Figure S10: (a) Height distribution histograms for all samples, (b) Variation of Skewness (▲, left axis) and Excess Kurtosis (⬤, right axis) across the sample set. The black dotted line corresponds to a balanced distribution between peaks and valleys, while the red dotted line at zero indicates a perfect Gaussian distribution.
