# Supplementary material for: Conductive Polyaniline-Based/Polyethersulfone Ultrafiltration Membranes: Morphology, Wettability and Short-Cycle Electrochemical Cleaning
Source: Membranes (Basel). 2026 Jun 3;16(6):194. doi: 10.3390/membranes16060194 (PMC13304109; doi:10.3390/membranes16060194)
Supplement: Supplementary file 1 [file membranes-16-00194-s001.zip › membranes-4263080-supplementary.pdf]

Conductive PAni·DBSA‑PES Ultrafiltration Membranes: Morphology, Wettability and Short‑Cycle Electrochemical Cleaning

Maria Antonia Rodrigues De Paulo[1], Roger Gonçalves[1], Fernando Henrique Cristovan[2], Adriana Coatrini Thomazi[3], Ernesto Chaves Pereira[1], José Arnando Santana Costa[4] and Caio Marcio Paranhos[1,].*

[1]CDMF, Department of Chemistry, Federal University of São Carlos, 13565-905 São Carlos, São Paulo, Brazil

[2]Institute of Exact Sciences and Technology, Federal University of Jataí, 75801-615 Jataí, Goiás, Brazil

[3] National Nanotechnology Laboratory for Agriculture (LNNA), Embrapa Instrumentation, XV de Novembro ST., 1452, São Carlos 13560-970, SP, Brazil

[4]GENE, Institute of Education Sciences, Federal University of Western Pará, 68040070, Santarém, Pará, Brazil.

*Corresponding Author: Tel: +55 16 3351-8080, email: paranhos@ufscar.br (Caio Marcio Paranhos)

Table S1. Membrane code and composition.

Sample	Matrix	PAni (%)
M1	PES	0
M2	PES/PAni.DBSA	0.1
M3	PES/PAni.DBSA	0.2
M4	PES/PAni.DBSA	0.5
M5	PES/PAni.DBSA	1.0
M6	PES/PAni.DBSA	2.0

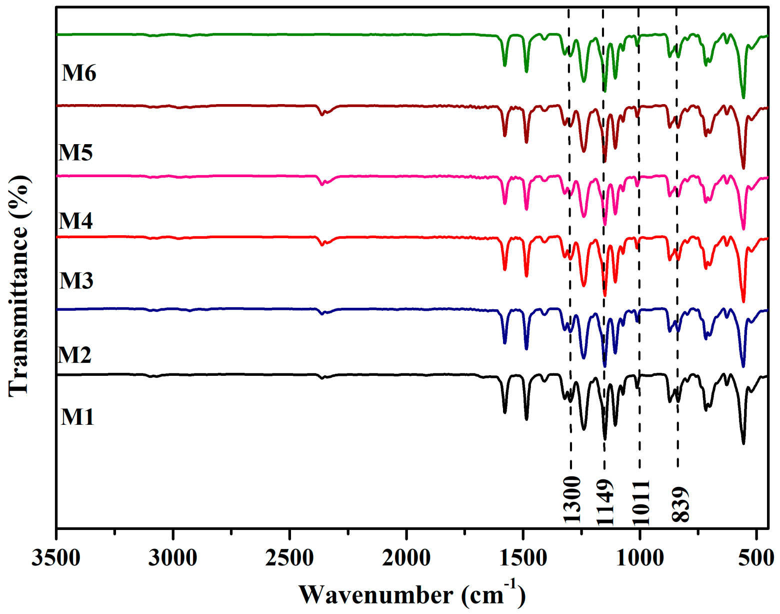

Figure S1 – FTIR spectrum of the electromembranes

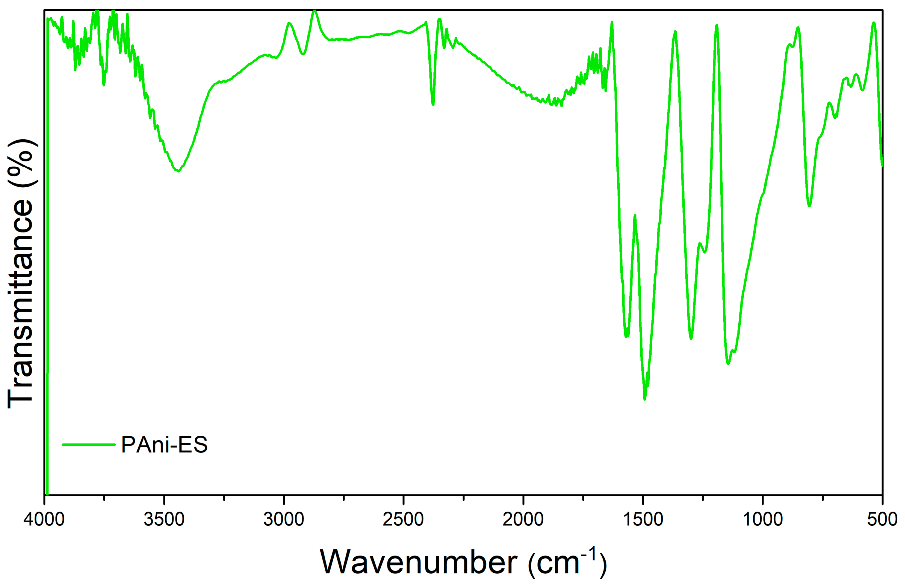

Figure S2: FTIR spectrum of PAni.

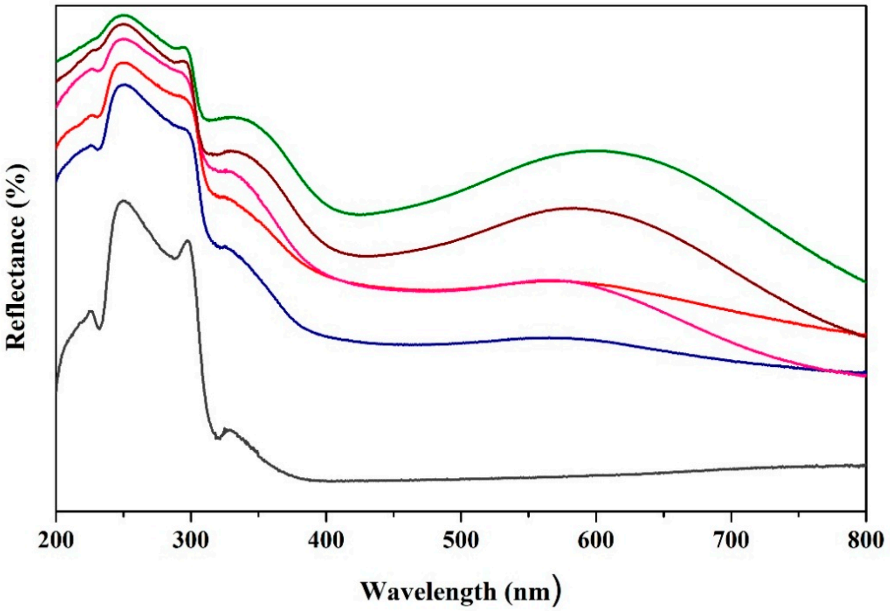

Figure S3. UV-vis spectra of the membranes :(—) PES; (—) M2; (—) M3; (—) M4; (—) M5; (—) M6.

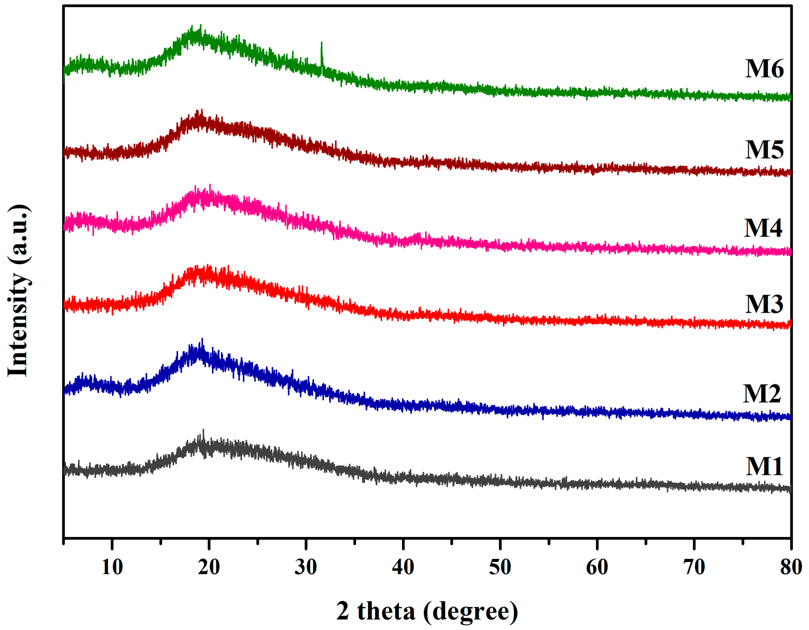

Figure S4: XRD patterns of membranes.

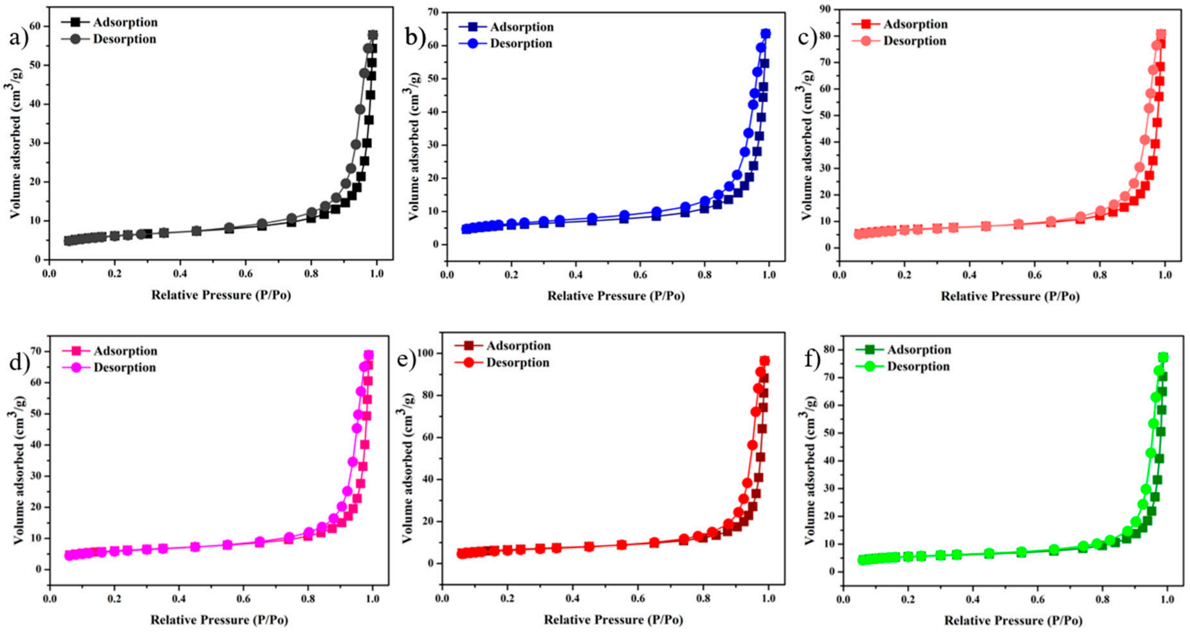

Figure S5. Nitrogen adsorption-desorption isotherms for membranes: a) M1; b) M2; c) M3; d) M4; e) M5; f) M6.

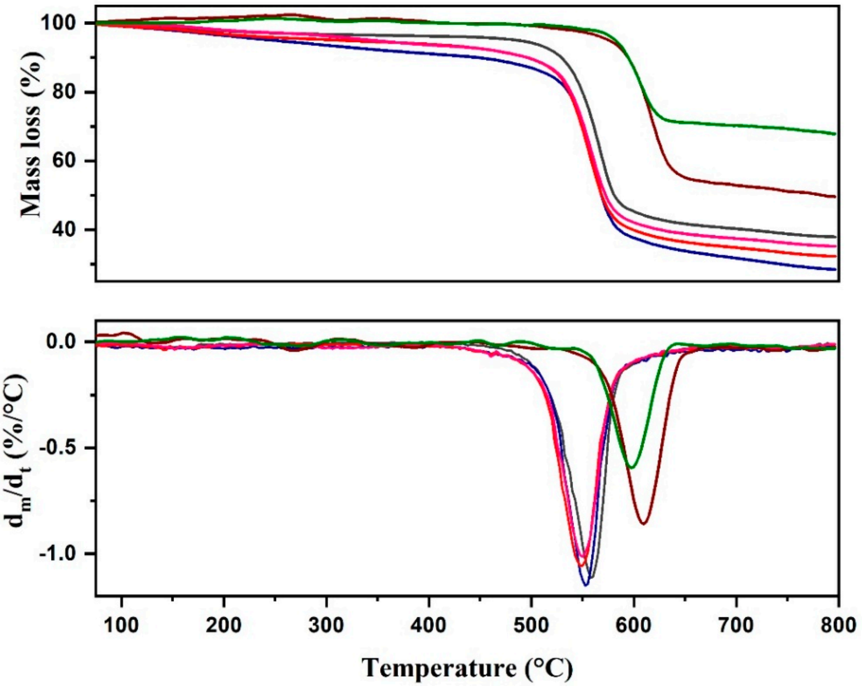

Figure S6. Thermogravimetric analysis for membranes: (—) M1; (—) M2; (—) M3; (—) M4; (—) M5; (—) M6.

AFM images analysis

Atomic Force Microscopy (AFM) was employed to characterize the topographical changes resulting from PAni.DBSA incorporation. The 2D top-down images, presented in Figure S7, provide a direct visualization of the surface texture and feature distribution. The control membrane, M1 (Figure S7a), shows a relatively uniform and featureless surface. In contrast, the addition of PAni induced clear morphological changes. The nodular, "brain-like" texture of M2 is evident (Figure S7b), while M3 (Figure S7c) displays large, broad features indicative of high heterogeneity. The M4 surface (Figure S7d) appears very smooth, distinguished only by a localized scratch, and M6 (Figure S7f) is characterized by bright, scattered spots corresponding to surface peaks.

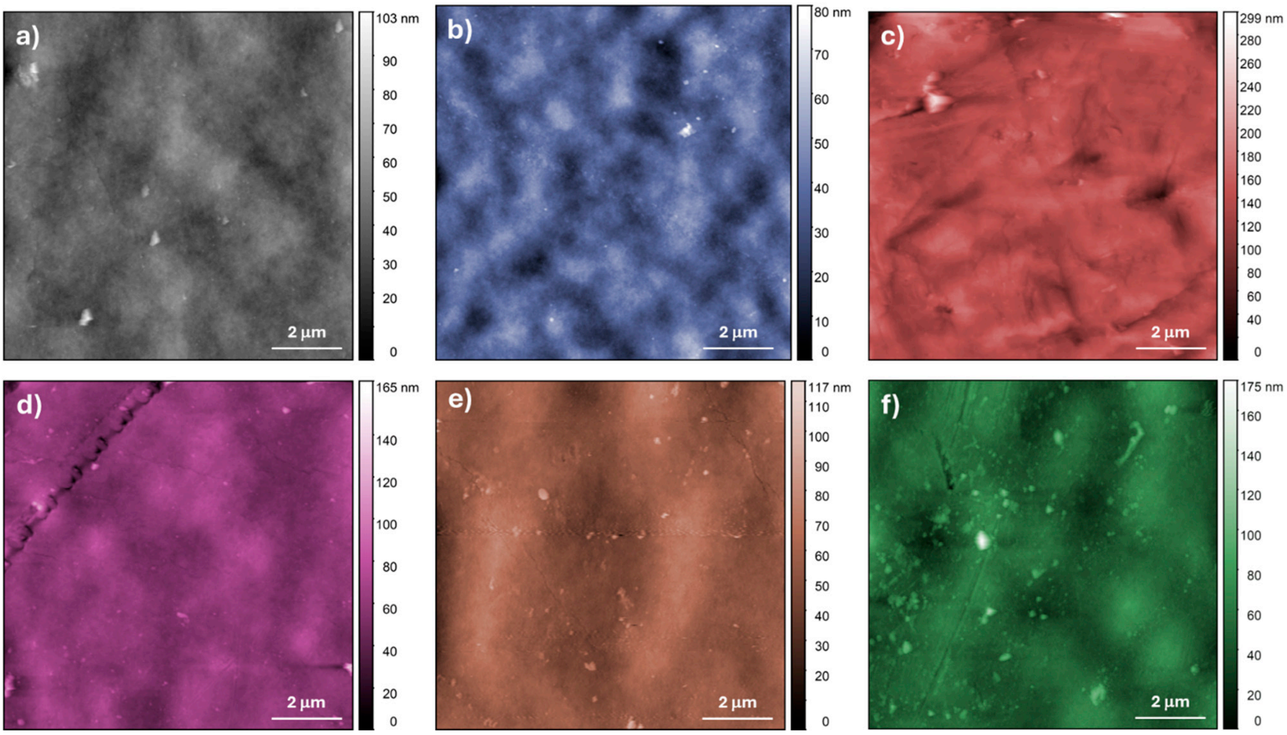

Figure S7: AFM 2D topographies (10 µm × 10 µm). z-range: M1 0–103 nm; M2 0–80 nm; M3 0 – 299 nm; M4 0 – 165 nm; M5 0 – 117 nm; M6 0 – 175 nm; N = 3 areas per sample."

The 3D reconstructions of the topography (Figure S6) offer a clearer perspective on the height variations and the amplitude of these features. The large, mountain-like structures of M3 are visually confirmed (Figure S8c), corresponding to its significant roughness. The 3D view of M6 (Figure S8f) reveals that its features are sharp, prominent peaks rising from the surface. Conversely, the nodular texture of M2 is shown to be composed of rounded hills and valleys (Figure S8b), while the overall flatness of the M4 surface is emphasized (Figure S8d).

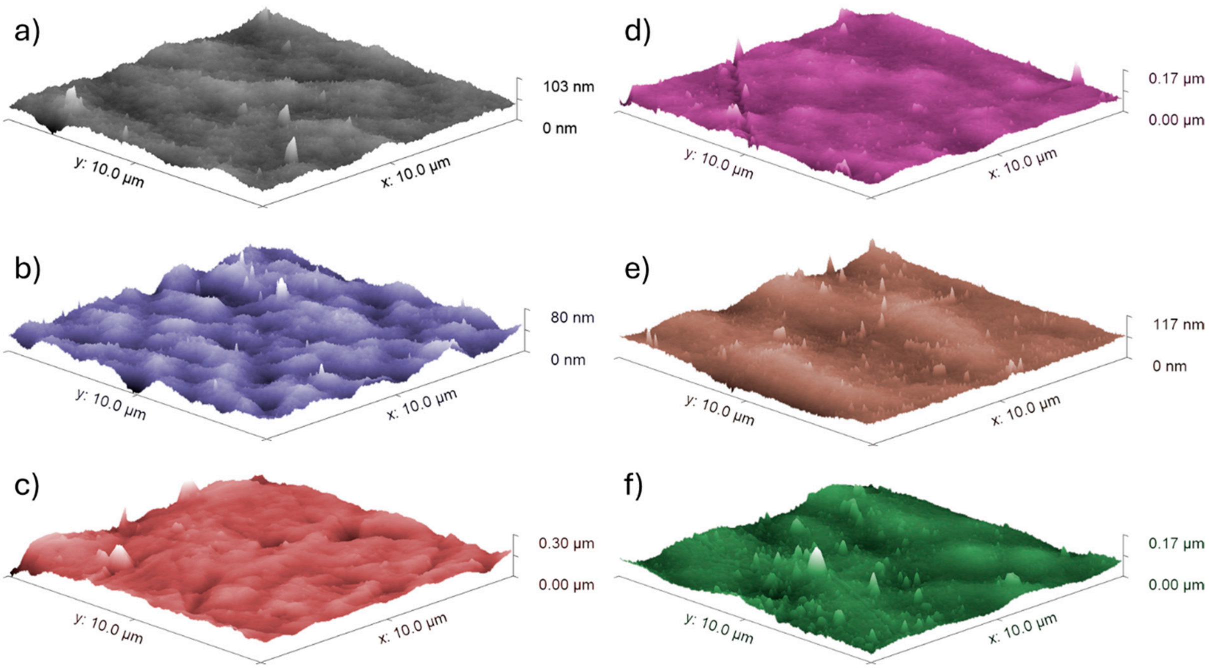

Figure S8: 3D topographical AFM images (10 µm x 10 µm) of the membrane surfaces. Images correspond to samples (a) M1, (b) M2, (c) M3, (d) M4, (e) M5, and (f) M6. The z-axis scale indicates the height range for each sample.

To quantify these visual observations, roughness parameters were calculated from the topographical data, as summarized in Table S2. The pristine PES membrane (M1) had an R_q of 6.78 nm. The incorporation of PAni.DBSA induced significant changes in roughness, which peaked for sample M3 (0.2% PAni) with an R_q of 18.1 nm. This peak roughness coincides with the highest flux recovery rate (FRR) and one of the best BSA rejection performances found in the filtration tests, suggesting a complex and beneficial relationship between this specific topography and the membrane's anti-fouling behavior.

Table S2: Surface roughness and topography parameters obtained from AFM analysis.

Sample	Rq (nm)	Ra (nm)	R_q / R_a	Skewness	Excess kurtosis
M1	6.78 ± 1.2	5.26 ± 1.0	1.29	0.610 ± 0.016	0.560 ± 0.25
M2	7.60 ± 1.1	6.10 ± 1.0	1.25	-0.402 ± 0.068	-0.699 ± 0.25

M3	18.1 ± 1.8	13.3 ± 1.4	1.36	-0.227 ± 0.012	2.25 ± 0.90
M4	7.61 ± 1.4	5.79 ± 1.2	1.31	0.0366 ± 0.0084	1.65 ± 0.31
M5	7.63 ± 1.5	5.95 ± 1.4	1.28	0.294 ± 0.067	0.737 ± 0.030
M6	11.7 ± 1.8	8.99 ± 1.4	1.31	0.500 ± 0.068	0.787 ± 0.21

The roughness data is further illustrated in Figure S9. The bar chart (Figure S9a) provides a clear visual comparison of the R_a and R_q values, emphasizing that M3 is more than twice as rough as the control membrane. The average surface line profiles (Figure S9b) offer additional insight into the cross-sectional nature of these textures. The profile for M3 confirms its high roughness, exhibiting the largest peak-to-valley amplitude. The M6 profile is characterized by sharp, sporadic peaks rising from a flatter baseline, consistent with its peak-dominated morphology. In contrast, the profile for M2 displays a more regular, wavy pattern that reflects its nodular structure.

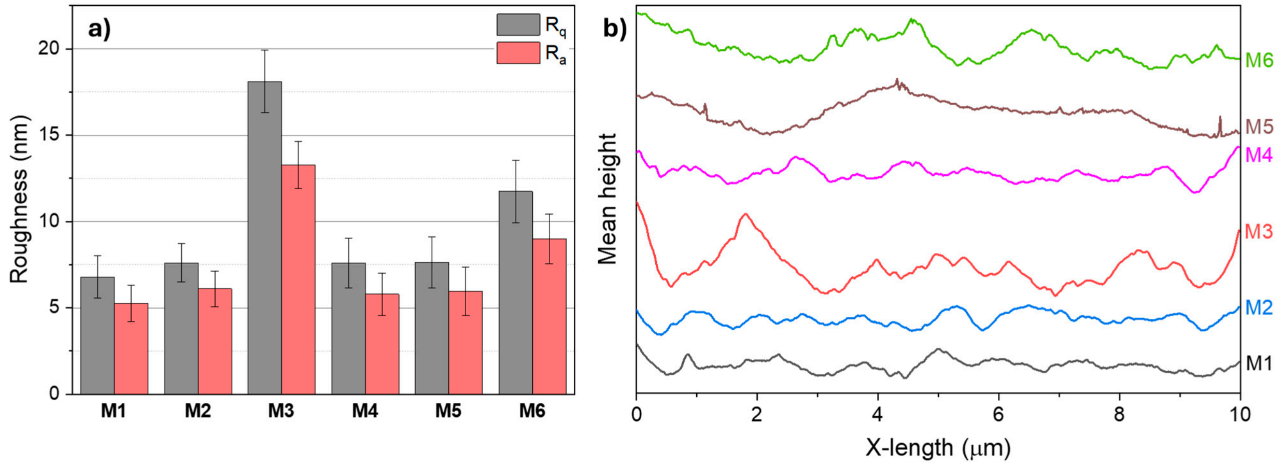

Figure S9: a) Comparison of R_q and R_a values for samples, b) Average surface line profiles extracted from the AFM topographical images, illustrating the characteristic cross-sectional texture of each membrane.

Beyond overall roughness, the distribution of surface heights, characterized by skewness and excess kurtosis, is crucial for understanding the varying performance of membranes with similar R_q values, such as M2 and M4. The height distribution histograms are presented in Figure S10a. The significantly broader distribution for M3 provides direct visual confirmation of its large R_q, while the shape of the curves offers a unique topographical fingerprint for each surface. The variation of these shape parameters is plotted in Figure S8. The skewness trend highlights M2 as the only sample with a strongly valley-dominated topography (negative skewness). The excess kurtosis data further distinguishes the surfaces, confirming that M3 and M4 have leptokurtic (spiky) profiles, while M2 has a platykurtic (bumpy) profile. This detailed information explains why M2 and M4, despite having nearly identical R_q values, exhibit vastly different flux recovery rates.

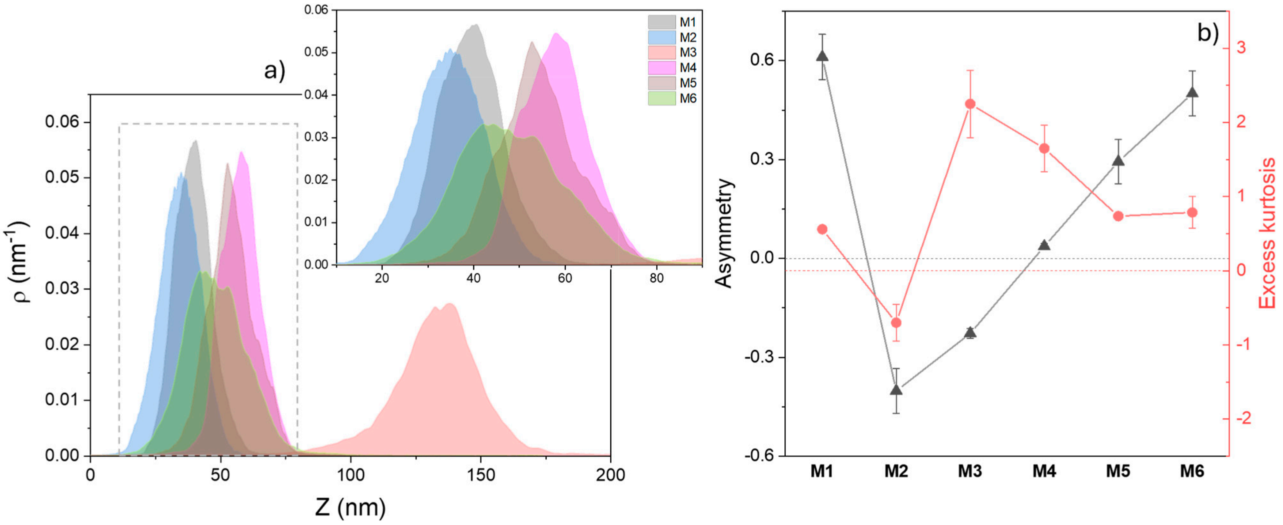

Figure S10: a) Height distribution histograms for all samples, b) Variation of Skewness (▲, left axis) and Excess Kurtosis (●, right axis) across the sample set. The black dotted line corresponds to a balanced distribution between peaks and valleys, while the red dotted line at zero indicates a perfect Gaussian distribution.

The Average Roughness (R_a) represents the arithmetic mean of the absolute height deviations from the mean plane. This parameter provides a general estimate of the surface texture but is less sensitive to extreme peaks or valleys. On the other hand, Root Mean Square Roughness (R_q)

corresponds to the standard deviation of the height distribution. By giving greater weight to large deviations, the R_q value is more sensitive to sharp peaks and valleys, making it a statistically more robust measure of overall roughness. Thus, presenting both is essential for a comprehensive surface characterization, as they respectively capture the average texture and the influence of extreme topographical features. Additionally, it is possible to use Skewness (or asymmetry, R_{sk}), which measures the degree of symmetry of the surface profile. Positive values ($R_{sk} > 0$) indicate a surface dominated by peaks and aggregates, while negative values ($R_{sk} < 0$) suggest a predominance of valleys and pores. An R_{sk} value close to zero characterizes a symmetrical height distribution.

Excess Kurtosis describes the sharpness or "peakedness" of the height distribution relative to a perfect Gaussian curve, which has an excess kurtosis of 0. Positive values (> 0) indicate a spiky (leptokurtic) distribution, characteristic of surfaces with an abundance of extreme peaks and valleys. Conversely, negative values (< 0) represent a flatter (platykurtic) distribution, suggesting that surface heights are more uniformly distributed without significant peaks or valleys.

The AFM analysis provides a multi-faceted view of the membrane topography. The integration of visual analysis (Figures S7 and S8), quantitative roughness data (Table S2 and Figure S9), and height distribution data (Figure S10) demonstrates that PAni.DBSA incorporation controls not just the magnitude of roughness but the specific nature of the surface texture. It is this detailed topographical fingerprint that, in synergy with the material's chemical properties, dictates the complex anti-fouling and filtration performance of the membranes.

Table S3 – Electrical conductivity and Fof PES and PES/Pani membranes.

Sample	Electric conductivity (10^{-8} S.cm^{-1})
M1	8.4 ± 0.1
M2	6.8 ± 0.4
M3	1.5 ± 0.01
M4	5.5 ± 0.3
M5	6.3 ± 0.7
M6	3.6 ± 0.3

Table S4 – Absolute FRR values obtained for PES and PAni-based membranes

Sample	Absolute FRR (%)
M1	58.3 ± 11.1
M2	442.6 ± 87.2
M3	473.3 ± 21.7
M4	188.0 ± 10.9
M5	172.8 ± 10.5
M6	224.3 ± 40.6

Table S5- Data correlation between several aspects of the membranes.

Sample	Specific Surface Area (m^2/g)	Pore Volume (cm^3/g)	Pore Diameter Range (µm)	Ra (nm)	Contact angle	J (ml.cm^{-2}.h^{-1})	RR(%)	FRR	J (ml.cm^{-2}.h^{-1})	RR(%)
M1	22.40	0.07		5.26	77.35	0.59	72.30	1	1.02	87.15

M2	21.04	0.07	0.70	6.10	64.78	0.56	76.46	7.59		
M3	24.73	0.10	0.30	13.3	63.88	0.95	78.50	8.12	6.65	99.32
M4	20.97	0.08	0.12	5.79	57.15	3.93	78.05	3.22	3.77	98.87
M5	23.75	0.11	3.4	5.95	55.31	3.86	76.47	2.96		
M6	19.71	0.09	0.50	8.99	69.45	2.41	75.08	3.85		
